# Sinking Skin Flap Syndrome following Posttraumatic Hydrocephalus

**DOI:** 10.1155/2021/6682310

**Published:** 2021-02-09

**Authors:** Ashish Chugh, Prashant Punia, Sarang Gotecha

**Affiliations:** Dr. D. Y. Patil Medical College and Hospital, Pimpri, Pune, Maharashtra, India

## Abstract

**Introduction:**

Complications following craniotomy are not uncommon and Sinking Skin Flap Syndrome (SSFS) constitutes a rare entity that may present after a large Decompressive Craniectomy. Although the entity is widely reported, the literature mostly consists of case reports. Authors present a case series of three patients with review of literature highlighting the various factors which can prove therapeutic and can help in avoidance of complications.

**Materials and Methods:**

The study was conducted over a period of 3 years, from 2016 to 2019, and included 212 patients who underwent unilateral Decompressive Craniectomy (DC) for trauma in our institute. All 212 patients underwent a similar DC following a strict institutional protocol and the craniectomies were performed by the same surgical team. At total of 160 patients survived and elective cranioplasty was planned at a 3-month interval. Out of a total of 160 patients who survived, 38 developed hydrocephalus, 3 patients presented with hydrocephalus acutely and had to be shunted before cranioplasty and underwent ventriculoperitoneal (VP) shunting on the opposite side of craniectomy. All 3 of these patients developed SSFS and were the focus of this case series wherein review of literature was done with emphasis being laid on the salient features towards management of SSFS in such precranioplasty shunted patients. These 3 patients were treated via rehydration using normal saline (NS) till the Central Venous Pressure (CVP) equaled 8–10 cm of water, nursing in Trendelenburg position and shunt occlusion using silk 3-0 round bodied suture tied over a “C”-loop of VP shunt tube over clavicle. This was followed by cranioplasty within 2 days of presentation using a flattened, nonconvex artificial Polymethyl Methacrylate (PMMA) bone flap with central hitch suture taken across the bone flap and release of shunt tie in immediate postoperative period. The PMMA bone flap was made intraoperatively after measuring the defect size accurately after exposure of defect. 3D printing option was not availed by any patient considering the high cost and patients' poor socioeconomic status.

**Results:**

Out of a total of 212 patients, thirty-eight patients (19%) developed posttraumatic hydrocephalus and out of 38, three presented with SSFS over the course of time. Two patients presented with hemiparesis of the side opposite to sunken flap while 1 other patient was brought by relatives in stuporous state. All 3 were subjected to VP shunt tie, rehydration, and cranioplasty using flattened artificial bone flap and showed gradual recovery in postoperative period without any complications.

**Conclusion:**

Various factors like nursing in Trendelenburg position, adequate rehydration, early cranioplasty after resolution of oedema, preoperative tying of VP shunt and its subsequent release in immediate postoperative period, use of flattened PMMA bone flaps, placement of a central dural hitch suture across the bone, and a preoperative central burr hole in the bone flap may accelerate healing and, in most cases, reversal of sensory-motor deficits along with reduction in complication rates.

## 1. Introduction

Complications following craniotomy are not uncommon and Sinking Skin Flap Syndrome (SSFS) constitutes a rare entity that may present after a large Decompressive Hemicraniectomy (DC) [1]. Also known by other names such as syndrome of the “trephined,” it consists of sunken skin above the bone defect along with neurological symptoms which can be but are not limited to headaches, motor and sensory deficits, mental changes, and seizures. Although the entity is widely reported, the literature mostly consists of case reports. Authors present a case series of three patients with review of literature highlighting the various factors which can prove therapeutic and can help in avoidance of complications.

## 2. Materials and Methods

The study was conducted over a period of 3 years, from 2016 to 2019, and included 212 patients who underwent unilateral Decompressive Craniectomy (DC) for trauma in our institute. All 212 patients underwent a similar DC following a strict institutional protocol and the craniectomies were performed by the same surgical team. At total of 160 patients survived and elective cranioplasty was planned at a 3-month interval.

Out of a total of 160 patients who survived, 38 developed hydrocephalus and 17 of these 38 patients were managed perioperatively during cranioplasty with ventricular/lumbar drain, 6 other patients denied any further treatment and left against medical advice, and 5 patients developed hydrocephalus after cranioplasty and were shunted within 6 weeks from cranioplasty with no complications. A total of 7 patients were lost to follow-up while the remaining 3 patients presented with hydrocephalus acutely and had to be shunted before cranioplasty and underwent ventriculoperitoneal (VP) shunting on the opposite side of craniectomy. These 3 patients were shunted with a low pressure VP shunt after measuring the opening pressure of the ventricle tap. Cranioplasty was not chosen to be done alongside VP shunting to avoid the added risk of infection.

All 3 of these patients developed SSFS and were the focus of this case series wherein review of literature was done with emphasis being laid on the salient features towards management of SSFS in such precranioplasty shunted patients.

## 3. Procedures

### 3.1. Decompressive Craniectomy

On the affected side, a question mark/reverse question mark incision was made 2 cms from midline with a limb length of 15 cms reaching zygoma. Craniectomy was done in a standard and uniform fashion with 4 burr holes each at the apex of bony exposure. Burr holes were connected and temporal bone was nibbled away in the region of zygoma. The standard sized craniotomy provided a uniform and adequate decompression. Hemostasis was achieved and incision was closed in 2 layers.

### 3.2. Cranioplasty for SSFS

These 3 patients were treated via rehydration using normal saline (NS) till the Central Venous Pressure (CVP) equaled 8–10 cm of water, nursing in Trendelenburg position, and shunt occlusion using silk 3-0 round bodied suture tied over a “C”-loop of VP shunt tube over clavicle. This was followed by cranioplasty within 2 days of presentation using a flattened, nonconvex artificial Polymethyl Methacrylate (PMMA) bone flap with central hitch suture taken across the bone flap and release of shunt tie in immediate postoperative period. The PMMA bone flap was made intraoperatively after measuring the defect size accurately after exposure of defect. 3D printing option was not availed by any patient considering the high cost and patients' poor socioeconomic status.

## 4. Case Series

### 4.1. Case 1

A 55-year-old female patient who sustained head trauma in a Road Traffic Accident (RTA) and underwent left frontotemporoparietal decompression craniectomy was brought by relatives with deteriorated neurological status (GCS-9) after 2 months. Examination revealed a tense scalp skin flap at the craniectomy site and the patient was rushed to CT scan which revealed hydrocephalus. Ventriculoperitoneal shunt (VP shunt) on the right side was done in emergency setting and the patient gradually progressed to her preoperative neurological status and was discharged.

**Figure 1:**
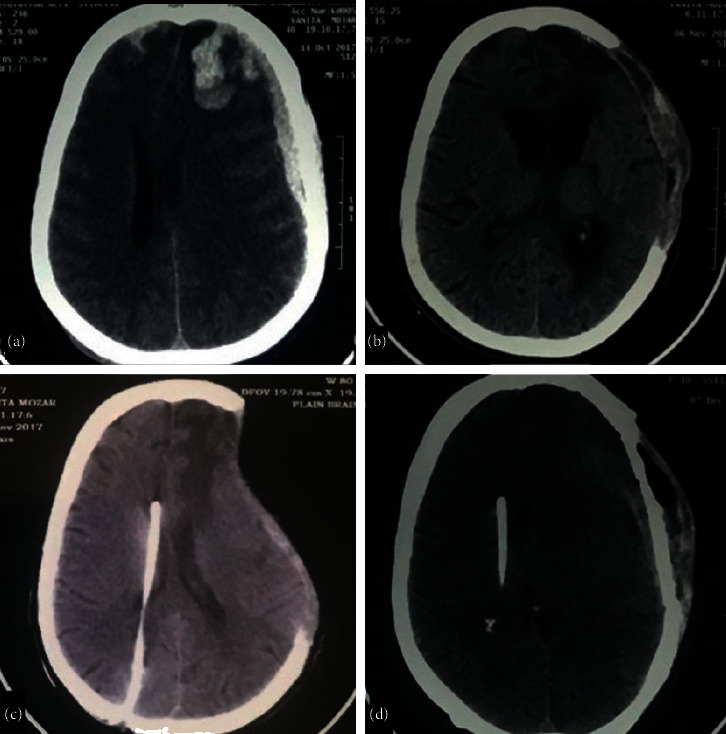
(a) Computed tomography (CT) plain brain, axial view showing hyperdense collection along left subdural plane extending across frontal, temporal, and parietal regions. (b) Computed tomography (CT) plain brain, axial view showing dilated ventricles and herniation of brain from craniectomy site. (c) Computed tomography (CT) plain brain, axial view showing VP shunt in situ and excessively sunken brain at craniectomy site causing midline shift to right side. (d) Computed tomography (CT) plain brain, axial view on postoperative day 1, showing cranioplasty using flattened bone. Also, partial resolution of midline shift can be seen.

The patient again presented 2 months later with right side spastic hemiparesis and severely sunken skin flap on the operated site. A diagnosis of SSFS was made and cranioplasty was planned. The patient was positioned in Trendelenburg position and rehydrated using NS and shunt was tied at the level of clavicle using silk 3-0 round bodied suture after making a C-loop of shunt so that it can be safely removed after cranioplasty. In 2 days, the flap was spontaneously significantly elevated and cranioplasty was performed with a flattened, artificial PMMA bone flap. Shunt occlusion was released immediately postoperatively and the patient gradually fully recovered her hemiparesis and was discharged on the 7th postoperative day (Figure 1).

### 4.2. Case 2

**Figure 2:**
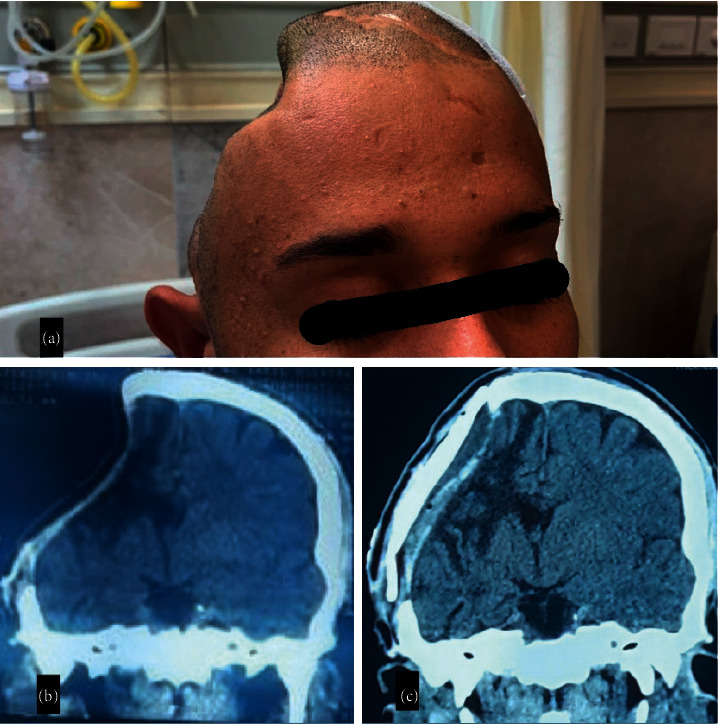
(a) Clinical photograph showing sunken skin flap 3 months after craniectomy. (b) Computed tomography (CT) plain brain, coronal view showing right-sided craniectomy with severely sunken hemisphere on the same side. (c) Computed tomography (CT) plain brain, coronal view on postoperative day 1, showing cranioplasty using flattened bone. Also, partial resolution of pressure effects can be seen.

A 22-year-old male patient who had undergone a decompression craniotomy on the right side for a RTA 1 month back presented with sudden deterioration on consciousness and headache. CT brain revealed hydrocephalus and patient underwent VP shunt procedure on the left side. Patient improved significantly after VP shunt and was discharged on the 7^th^ postoperative day; however, 2 months later, he presented to us in a stuporous state (GCS-9) with a severely sunken skin flap over the right side of the skull. The patient was rehydrated and nursed in Trendelenburg position for 2 days along with tying his VP shunt tube at the level of clavicle. Two days later, he underwent cranioplasty using a flattened, nonconcave artificial PMMA bone flap. Shunt tube block was removed in immediate postoperative period and the patient started showing improvement to be discharged on the 11^th^ postoperative day. Postoperative CT scan showed minimal subdural collection without any mass effect at the cranioplasty site which resolved on its own a few days later (Figure 2).

### 4.3. Case 3

**Figure 3:**
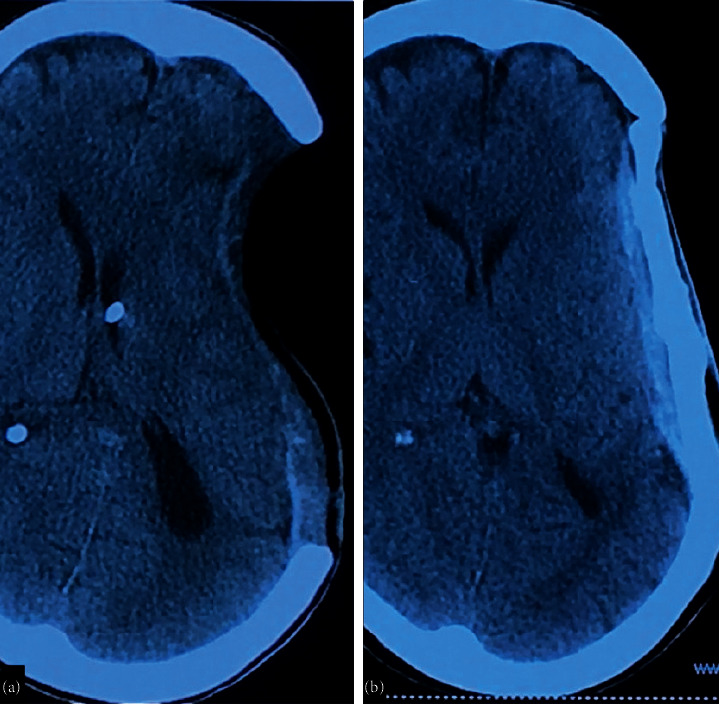
(a) Computed tomography (CT) plain brain, axial view showing VP shunt in situ and excessively sunken brain at craniectomy site causing midline shift to the right side. (b) Computed tomography (CT) plain brain, axial view on postoperative day 1, showing cranioplasty using flattened bone. Also, partial resolution of midline shift can be seen.

A 59-year-old female who underwent left decompression craniotomy for acute Subdural Hematoma (SDH) following RTA 1 month back presented to us with complaints of headache. She was investigated and CT brain plain was suggestive of communicating hydrocephalus for which the patient was posted for VP shunting in the right side. The patient's headache gradually improved in the early postoperative period and she was discharged on the 7^th^ postoperative day. Two months later, the patient was brought by relatives with complaints of a severely sunken skin flap over the skull and right hemiparesis. A diagnosis of SSFS was made and the patient was rehydrated and nursed in Trendelenburg position for 2 days along with tying her VP shunt tube in the anterior chest wall, at the level of clavicle. Patient underwent cranioplasty using flattened, nonconcave artificial PMMA bone flap and a central hitch suture over dura. Shunt block was removed immediately postoperatively and the patient showed gradual recovery to full power in her postoperative period. Postoperative CT scan showed good expansion of brain and partial resolution of midline shift and the patient was discharged on 15^th^ postoperative day with no deficits (Figure 3).

## 5. Discussion

Out of a total of 160 patients, thirty-eight patients (24%) developed posttraumatic hydrocephalus. The rate was slightly lower to 28% as observed by Nasi et al. [2] but was more than the rates reported by Yang et al. [3] who reported posttraumatic hydrocephalus in 17.6% of their patients. Rienzo et al. [4] in their prospective series of 269 patients of trauma reported an incidence of posttraumatic hydrocephalus in 34% of their patients which is comparable to our statistics.

A total of 21 out of 35 patients who developed hydrocephalus but did not progress to SSFS were males and 14 females whereas 2 out of 3 patients who developed SSFS were females. Across all major series, male predominance is seen and the reversal of this ratio in our series can be attributed to the limited size of 3 patients. All included patients in this series were operated on (DC) for trauma which has been seen as the leading cause for decompression craniotomy and is comparable with other larger studies [4]. In our series, in the non-SSFS group of 35 patients, 16 were operated on for Subdural Hematoma (SDH) alone while 11 others had SDH along with contusions. The remaining 8 patients were operated on for multiple contusions. In the SSFS group of 3 patients, 2 were diagnosed with SDH + contusions while 1 patient was operated on for SDH alone. Volume loss has been regarded as a potential causative factor towards SSFS and the same has been put across by Rienzo et al. [4] who stated that the brain's capacity to counterbalance the atmospheric pressure is significantly hampered when more brain tissue is lost [4].

As this case series is free of surgeon and surgery bias, the substantial difference between the patients not developing SSFS and those developing it remains preoperative VP shunting. It has been observed frequently that precranioplasty shunting leads to development of SSFS. This theory has been supported by Ashayari et al. [5] who stated that an increased gradient between atmospheric and intracranial pressures caused by CSF flow contributes to the development of SSFS. Although the pathophysiology of SSFS has never been adequately elucidated and the sample size of our series is too less to derive conclusions based on statistical analysis, it resonates with the published findings and it can be safely reiterated that precranioplasty shunting and an injury causing volume loss can be proposed as causative mechanisms.

Decompression Craniectomy (DC) is a life-saving procedure for patients presenting with raised intracranial pressure (ICP) due to various reasons. It renders the brain only with a skin cover which is desirable in the given circumstances; however, large cranial defects lead to ex-vacuo/compensatory dilatation and subsequent shifting of ipsilateral lateral ventricle resulting frequently in shunt dependent hydrocephalus [6]. The pathophysiology for development of posttraumatic hydrocephalus is poorly understood and it is attributed to the failure of arachnoid villi to absorb CSF as the craniectomy defect may lead to loss of normal waveform pattern of intracranial pressure (ICP) which, in turn, leads to loss of gradient which is essential to keep the villi open to absorb CSF.

Decompressive Craniectomy status, thus, leads to disturbances in CSF circulation and the same coupled with VP shunting and gradual decrease in pressure from inside due to resolving brain oedema aids in manifestation of detrimental effects of atmospheric pressure on the unsupported scalp. This leads to disturbances in arterial flow and venous return and the subsequent lowering of cortical perfusion in comparison to noncraniectomy hemisphere thus contributing to brain metabolism disorders [6]. Ashayeri et al. [5] defined 4 major factors responsible for development of SSFS viz atmospheric pressure, cerebral blood flow, CSF flow, and cerebral metabolism. They further stated that decreases in cerebral blood flow (CBF) have been shown and attributed the same to both atmospheric pressure and impairment of venous blood flow through the brain. These undesirable results may be compounded by posttraumatic brain atrophy or parenchymal volume loss on the affected side. This statement is further strengthened by the findings of Wee and Kuo [7], who stated that a VP shunt placement in patients with skull defect may lead to excessive sinking at the craniectomy site as a result of atmospheric pressure gradient, which can be aggravated by change in position.

Patients with SSFS who have a profound neurological deficit after DC have a favorable outcome after cranioplasty and the same has been observed across many case series and studies. Yamamura et al. [8] were one of the first to report clinical improvement after cranioplasty in patients with DC and depressed skin flap. Ashayeri et al. [5] in their series on SSFS stated that all of their patients showed some form of improvement with some patients showing improvement as early as 24 hours and at an average of 4 days. All 3 patients in our series started showing recovery in immediate postoperative phase and gradually recovered to preoperative status prior to discharge on the 7^th^ day.

Timing of cranioplasty has been an issue of debate and many authors recommend an early cranioplasty. The same has been described in various studies as that occurring between 1 and 6 months following DC. Songara et al. [9] in their prospective study on early cranioplasty observed significant improvement in GCS, Glasgow Outcome Scale (GOS), and Mini Mental State Examination (MMSE). They concluded that cranioplasty, when done early, is likely to restore the cerebral perfusion and CSF dynamics and decrease the complications as compared to late cranioplasty. Yoshida et al. [10] studied dynamics of cerebral blood flow and metabolism in patients with cranioplasty via spectroscopy and observed that cranioplasty should be carried out as soon as cerebral oedema has decreased because the defect in the bone by itself may cause decreased blood flow and disturbed metabolism. This view was seconded by Winkler et al. [11] who observed that that cranioplasty appears to increase blood flow velocity and cerebrovascular reserve capacity of both ipsilateral middle cerebral artery and internal carotid artery. Yang et al. [3] in their study tried to determine the timing of the earliest possible cranioplasty and concluded that cranioplasty can be considered as early as 34 days following DC. In authors' opinion, performing cranioplasty at a 3-month interval on the one hand gives adequate time for wound healing and recovery and at the same time saves the patient from deleterious effects of a late cranioplasty including SSFS. Malcolm et al. [12] concluded that cranioplasty might improve neurological function regardless of timing, but earlier cranioplasty may enhance this effect.

Many a times across developing countries, the patients do not turn up for regular follow-ups and protocols for timely cranioplasty cannot be ubiquitously applied. A total of 7 patients were lost to follow-up in our series and another 6 patients refused any further treatment due to poor socioeconomic status.

Also, delay in cranioplasty is seen in a subset of patients who present acutely with symptoms of raised intracranial pressure and neurological deterioration due to hydrocephalus before the planned 3-month interval, as seen with 3 of our patients. These patients were subjected to a VP shunting in emergency setting and cranioplasty was postponed to a later date. However, literature has multiple accounts stating that cranioplasty can itself lead to hydrocephalus resolution but at times the same is not feasible due the poor neurological status and tense craniectomy flap at presentation due to hydrocephalus and the surgeon is left with no option. The authors chose not to perform cranioplasty in the same sitting to prevent the risk of exposure of both CSF and flap contamination. Our view is shared by Di Rienzo et al. [4] who advocated that this practice of precranioplasty shunting should not be banished but should be tailored. They also agree with the authors' view on the increased risk of infection by performing dual procedures involving a foreign body.

After treatment of hydrocephalus, all 3 patients in our series presented with SSFS within 3 months and considerable doubt still remains on the time taken for SSFS to develop after VP shunting as the literature is scarce on such patients and further studies with a bigger sample size are needed to ascertain the same. Di Rienzo et al. [4] did not find a significant statistical correlation between timing of cranioplasty among patients with and without SSFS and even reported cranioplasty after 6 months from craniectomy in 16 patients with SSFS. The authors' view is that once SSFS is recognized, care must be taken not to delay the cranioplasty to the point of development of new deficits. Literature strongly stresses the relation between the use of shunts and occurrence of sinking as seen with 3 of our patients and the recognition of onset of the same should be taught to the patient/patient's relatives for quick recognition and action. This is sometimes delayed as these patients are often neglected and severely sunken flap along with motor weakness are the easily recognizable signs as compared to cognitive deficits [4]. Two of our patients were brought by relatives with hemiparesis and 1 was brought with a severely sunken flap.

### 5.1. Treatment

Performing a cranioplasty in a patient with severely convex flap has its demerits as it provides a potential dead space for accumulation of blood and/or fluids. Liao and Kao [13] concur with the authors and stated that a procedure for expansion of this dead space would be prudent. Han et al. [14] overcame this difficulty by reprogramming the shunt valve and temporarily increasing the valve pressure thus allowing expansion. This method may not be always possible especially in a developing country and thus tying and subsequent release of VP shunt is a cheap alternative with similar benefits. The shunt tie has to be over a bony prominence (clavicle) for easy recognition via palpation and shunt tube must be looped into “C” shape for hassle-free release of the tie after cranioplasty. All three of our patients were managed using shunt tie and its release in immediate postoperative period without any complications and the authors strongly recommend the same wherever a programmable shunt is not in place. Kim et al. [15] used the same technique with comparable results and stated that, apart from being a simple procedure without complications, it has other benefits too, like it can be done in the ward under local anaesthesia which is especially helpful to medically compromised patients wherein it saves the patient from multiple surgeries of shunt removal and subsequent insertion if needed.

Other strategies for expansion of the dead space are rehydration and putting the patient in Trendelenburg position. Rehydration is particularly helpful as these patients are usually chronically dehydrated. Both these measures were used in conjunction with shunt tie in our study as the effect of Trendelenburg position in increasing intracranial blood volume is limited as stated by Liao et al.

**Figure 4:**
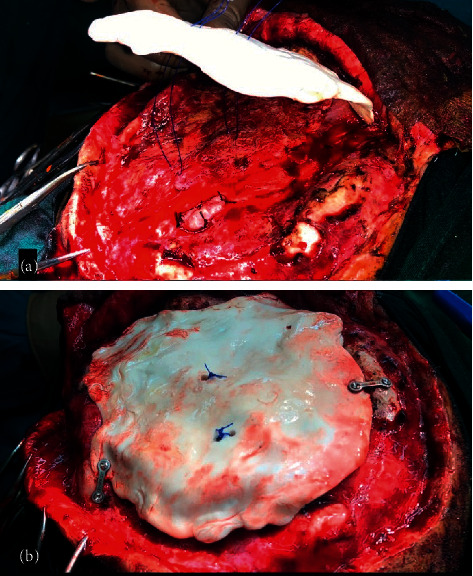
(a) Intraoperative photograph showing placement of central hitch suture across the flattened bone flap. (b) Intraoperative photograph after placement of central hitch suture across the bone flap.

The shape of bone flap plays an important role by itself in patients with SSFS as a convex/natural shaped flap provides extra potential dead space and thus, the authors used flattened bone flaps made with synthetic bone cement (PMMA) in all 3 of our patients. It was observed in late postoperative period that no appreciable change in skull shape was evident after full hair growth. The description on use of flattened bone flaps is limited in the available literature and because the current case series is limited to 3 cases, a detailed study is the need of the hour to prove the effectiveness of the same beyond doubt (Figure 4).

The material used for cranioplasty does not seem to play a role in postoperative complications and Lee et al. [16] demonstrated that the type of material used (PMMA vs. autologous) had no bearing on postoperative complications like extradural fluid collection (EFC). Moreover, Kim et al. [17] reported higher rates of EFC in autologous bone graft (61.1%) as compared to PMMA (43.2%). The authors, thus, used and recommend a flattened PMMA bone graft in patients with severely concave skin flaps, as in SSFS. This coupled with a central hitch suture put across the bone flap facilitated obliteration of extradural space and in turn decreases the chances of EFC.

Placement of a trephination burr hole Intra‐operatively at the center of the cranioplasty flap has been recommended by a few [18] and although the authors were not aware of the entity during the study time period, they can safely assert the obvious importance of the same in cases where postoperative collection is expected. Extradural fluid collection, if present, can safely be aspirated via this burr hole and this small step can prove vital in avoiding a resurgery.

## 6. Conclusion

Sinking Skin Flap Syndrome (SSFS) in patients after DC and VP shunting is not an uncommon entity in neurosurgical practice; yet, clear guidelines for treatment and avoidance of complications are not universal. The authors, with their own experience and after reviewing the relevant limited literature available, conclude that various factors like nursing in Trendelenburg position, adequate rehydration, early cranioplasty after resolution of oedema, preoperative tying of VP shunt and its subsequent release in immediate postoperative period, use of flattened PMMA bone flaps, placement of a central dural hitch suture across the bone, and a Intra‐operatively central burr hole in the bone flap may accelerate healing and, in most cases, reversal of sensory-motor deficits along with reduction in complication rates. Neurosurgeons across the globe should be well versed with the entity the and above said factors and their role; being forewarned is being forearmed.
